# Supplementary material for: Molecular mechanisms of thiazide-like diuretics-mediated inhibition of the human Na-Cl cotransporter
Source: Nat Commun. 2025 Aug 20;16:7740. doi: 10.1038/s41467-025-62714-w (PMC12365235; doi:10.1038/s41467-025-62714-w)
Supplement: Supplementary file 1 — Supplementary Information [file 41467_2025_62714_MOESM1_ESM.pdf]

SUPPLEMENTARY INFORMATION

Molecular mechanisms of thiazide-like diuretics-mediated inhibition of the human Na-Cl cotransporter

Chien-Ling Lee[1,#], Jianxiu Zhang[1,#], Liang Feng[1*]

[1]Department of Molecular and Cellular Physiology, Stanford University School of Medicine, Stanford, CA 94305, USA

[#]These authors contributed equally
[*]Correspondence to: liangf@stanford.edu

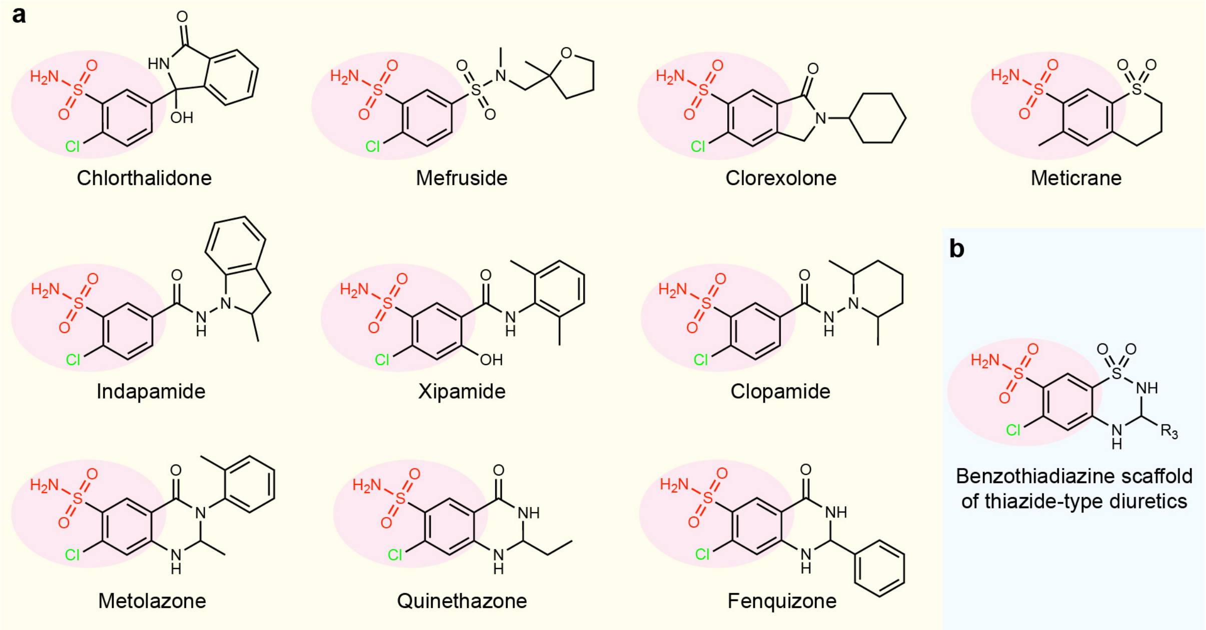

Supplementary Fig. 1 Chemical structures of thiazide diuretics. Thiazide-like diuretics (**a**) and the benzothiadiazine scaffold of thiazide-type diuretics (**b**) are shaded yellow and blue, respectively. The shared benzenesulfonamide moieties are highlighted with pink ovals. The sulfamoyl and chlorine groups are colored red and green, respectively.

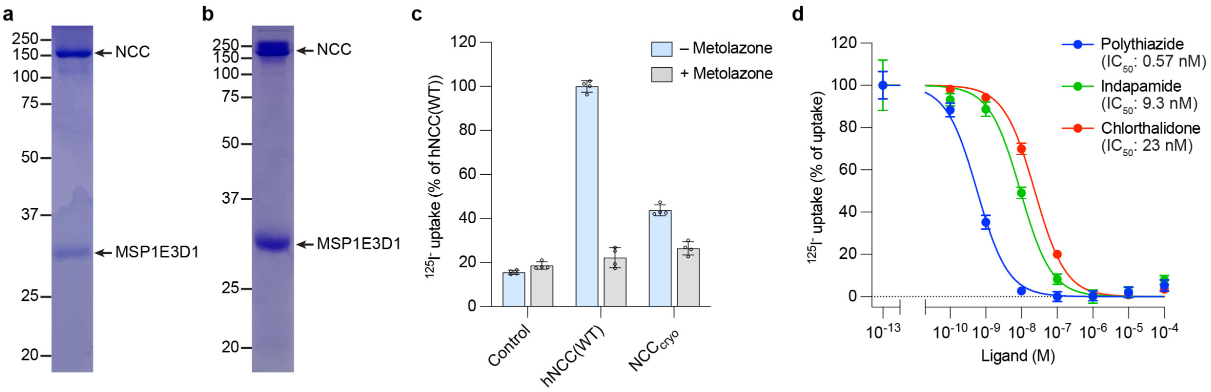

Supplementary Fig. 2 Cryo-EM sample preparation. a. and **b.** SDS-PAGE analysis of nanodisc reconstituted NCC_{cryo} copurified with chlorthalidone (**a**) and indapamide (**b**), respectively. **c.** NCC_{cryo} transport activity. For the metolazone groups, 100 μM metolazone was used. The values are normalized to that of hNCC(WT) in the absence of metolazone. Data are shown as mean ± s.d. (*n* = 4 independent experiments). **d.** Dose-response curves of different thiazide diuretics against hNCC(WT). Data are shown as mean ± s.d. (*n* = 3 independent experiments), with 1 mM extracellular iodide. Data for indapamide are the same as that for hNCC(WT) in Fig. 1g.

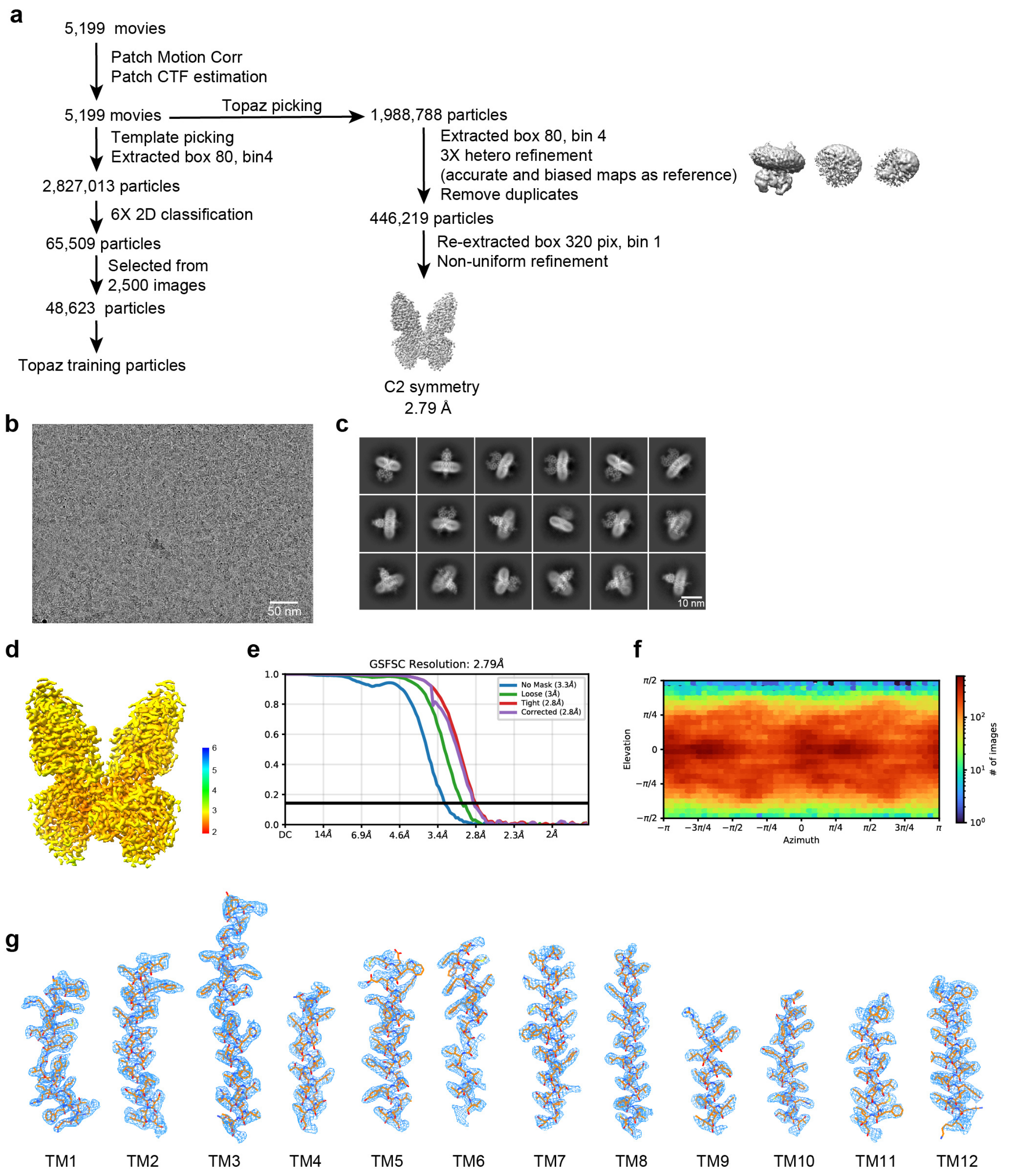

Supplementary Fig. 3 Cryo-EM data processing of indapamide-bound NCC_{cryo}. **a.** Workflow of indapamide-bound NCC_{cryo} data processing. **b.** A typical cryo-EM image. **c.** Typical 2D averages. **d.** Local resolution analysis of the indapamide-bound NCC_{cryo} map. **e.** Gold-standard Fourier shell correlation plot of the final whole map. **f.** Angular distribution of particles for the final map. **g.** Representative cryo-EM densities corresponding to the indapamide-bound NCC_{cryo} model (contour level: 5.7 σ).

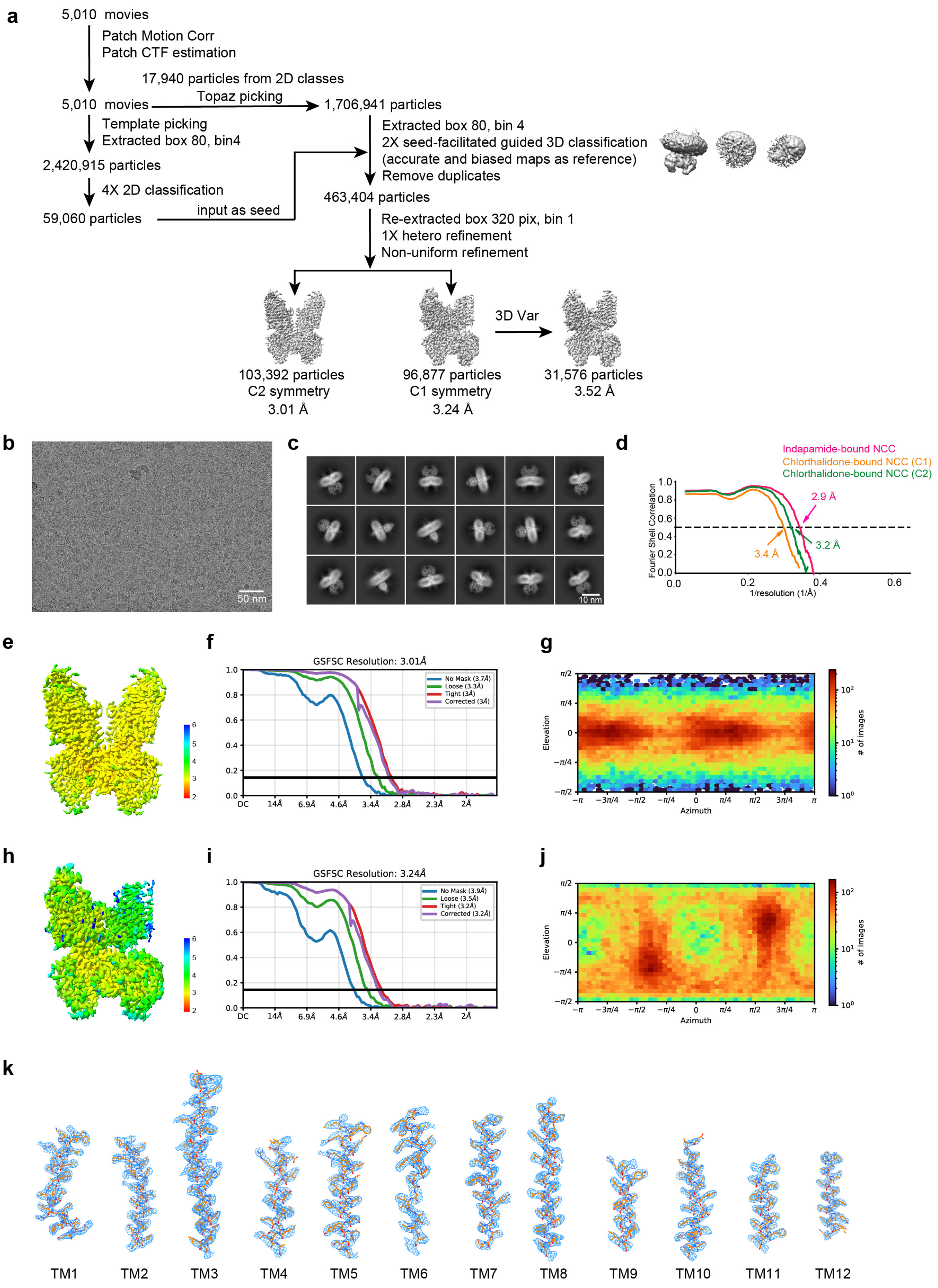

Supplementary Fig. 4 Cryo-EM data processing of chlorthalidone-bound NCC$_{cryo}$. **a.** Workflow of chlorthalidone-bound NCC$_{cryo}$ data processing. **b.** A typical cryo-EM image. **c.** Typical 2D averages. **d.** Map vs model FSC. **e.** and **h.** Local resolution

analysis of the symmetrical and asymmetrical chlorthalidone-bound NCC_{cryo} maps, respectively. **f.** and **i.** Gold-standard Fourier shell correlation plots of the final whole maps of the symmetrical and asymmetrical chlorthalidone-bound NCC_{cryo}, respectively. **g.** and **j.** Angular distribution of particles for the final maps of the symmetrical and asymmetrical chlorthalidone-bound NCC_{cryo}, respectively. **k.** Representative cryo-EM densities corresponding to the symmetrical chlorthalidone-bound NCC_{cryo} model (contour level: 6.7 σ).

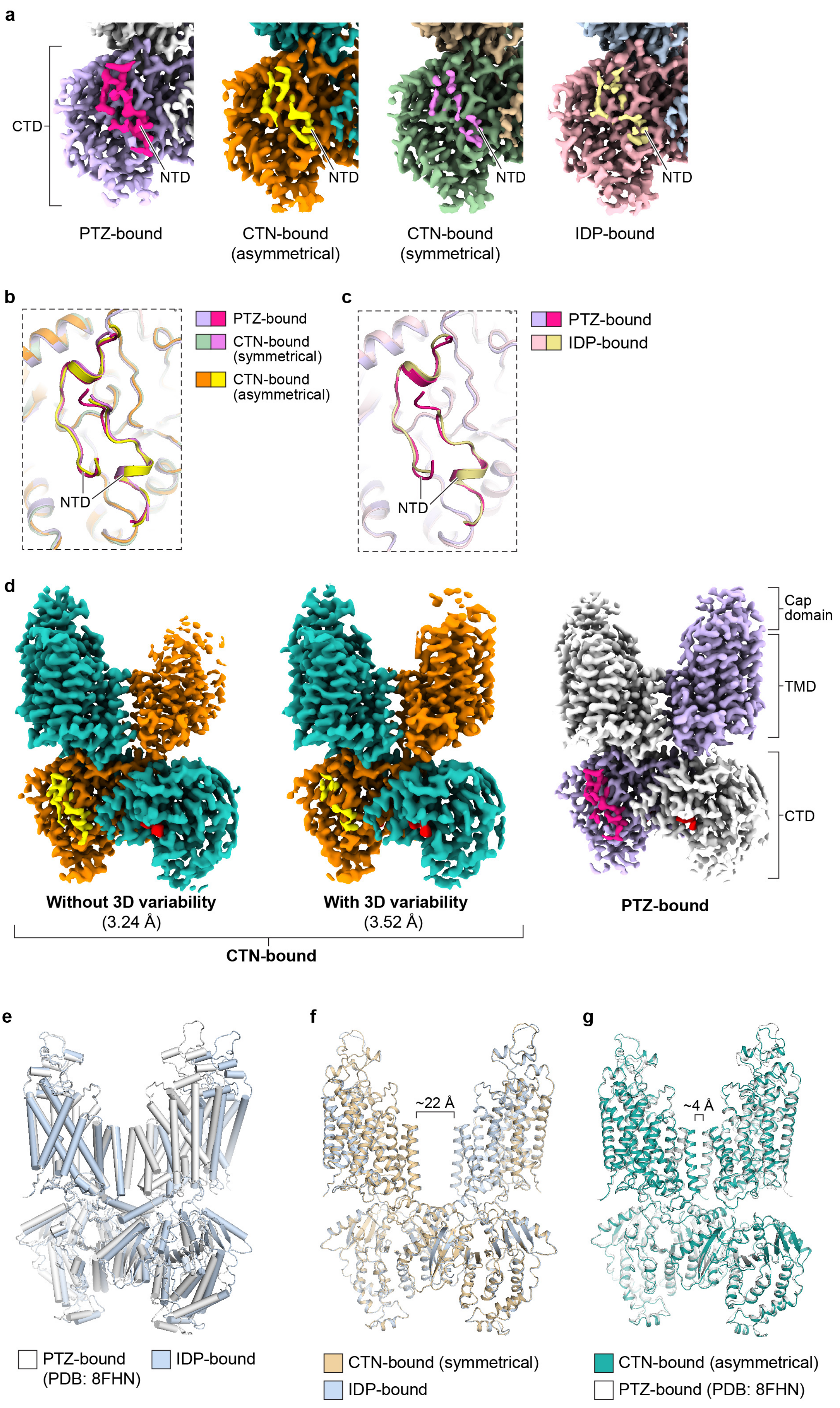
a
CTD
NTD
NTD
NTD
NTD
PTZ-bound
CTN-bound (asymmetrical)
CTN-bound (symmetrical)
IDP-bound
b
NTD
PTZ-bound
CTN-bound (symmetrical)
CTN-bound (asymmetrical)
c
NTD
PTZ-bound
IDP-bound
d
Cap domain
TMD
CTD
Without 3D variability (3.24 Å)
With 3D variability (3.52 Å)
CTN-bound
PTZ-bound
e
f
g
~22 Å
~4 Å
PTZ-bound (PDB: 8FHN)
IDP-bound
CTN-bound (symmetrical)
IDP-bound
CTN-bound (asymmetrical)
PTZ-bound (PDB: 8FHN)

Supplementary Fig. 5 Ligand-specific interdomain interactions. a. Comparisons of the NTD densities of NCC in complex with different thiazide diuretics. The PTZ-bound, asymmetrical and symmetrical CTN-bound, and IDP-bound maps are contoured at 4.9 σ, 6.9 σ, 5.4 σ, and 4.8 σ, respectively, to yield similar densities of the NTD-interacting CTDs. **b.** and **c.** Comparisons of the NTD-CTD interactions in NCC in complex with different thiazide diuretics. Different structures are aligned based on the NTD-interacting CTDs. **d.** 3D variability analysis of asymmetrical CTN-bound NCC. The PTZ-bound NCC map is included for comparisons of intradimer dynamics. The asymmetrical CTN-bound maps without and with 3D variability analysis and the PTZ-bound maps are contoured at 5.8 σ, 6.7 σ, and 4.9 σ, respectively, for comparable densities of the more CTD-interacting TMDs. In **a-d**, the color scheme is the same as in Fig. 1. **e.** Structural comparisons of the symmetrical (IDP-bound; light blue) and asymmetrical (PTZ-bound; white) NCC dimer configurations. The two structures are shown in cylindrical representations and aligned based on the more CTD-interacting TMD subunit. **f.** Comparisons of the IDP-bound (light blue) and symmetrical CTN-bound (tan) NCC structures. **g.** Comparisons of the PTZ-bound (white) and asymmetrical CTN-bound (teal) NCC structures. In **f** and **g**, the distances between TMD subunits are labeled.

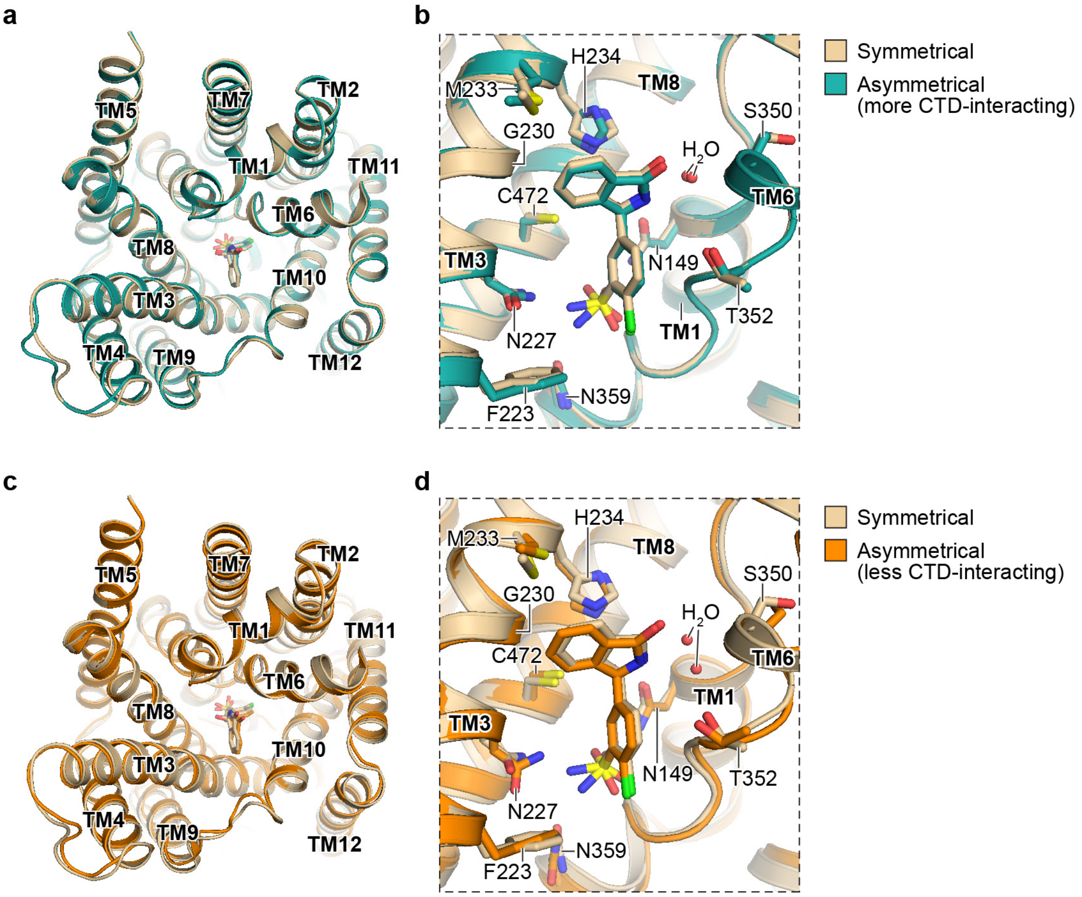

Supplementary Fig. 6 Comparisons between the TMDs of the symmetrical and asymmetrical CTN-bound NCC structures. The TMD of the symmetrical CTN-bound structure (tan) is compared to the more (teal) and less (orange) CTD-interacting TMD subunits of the asymmetrical CTN-bound structure in **a**,**b** and **c**,**d**, respectively. The cap domains are not shown for clarity. In **b** and **d**, TM10-12 are not shown for clarity.

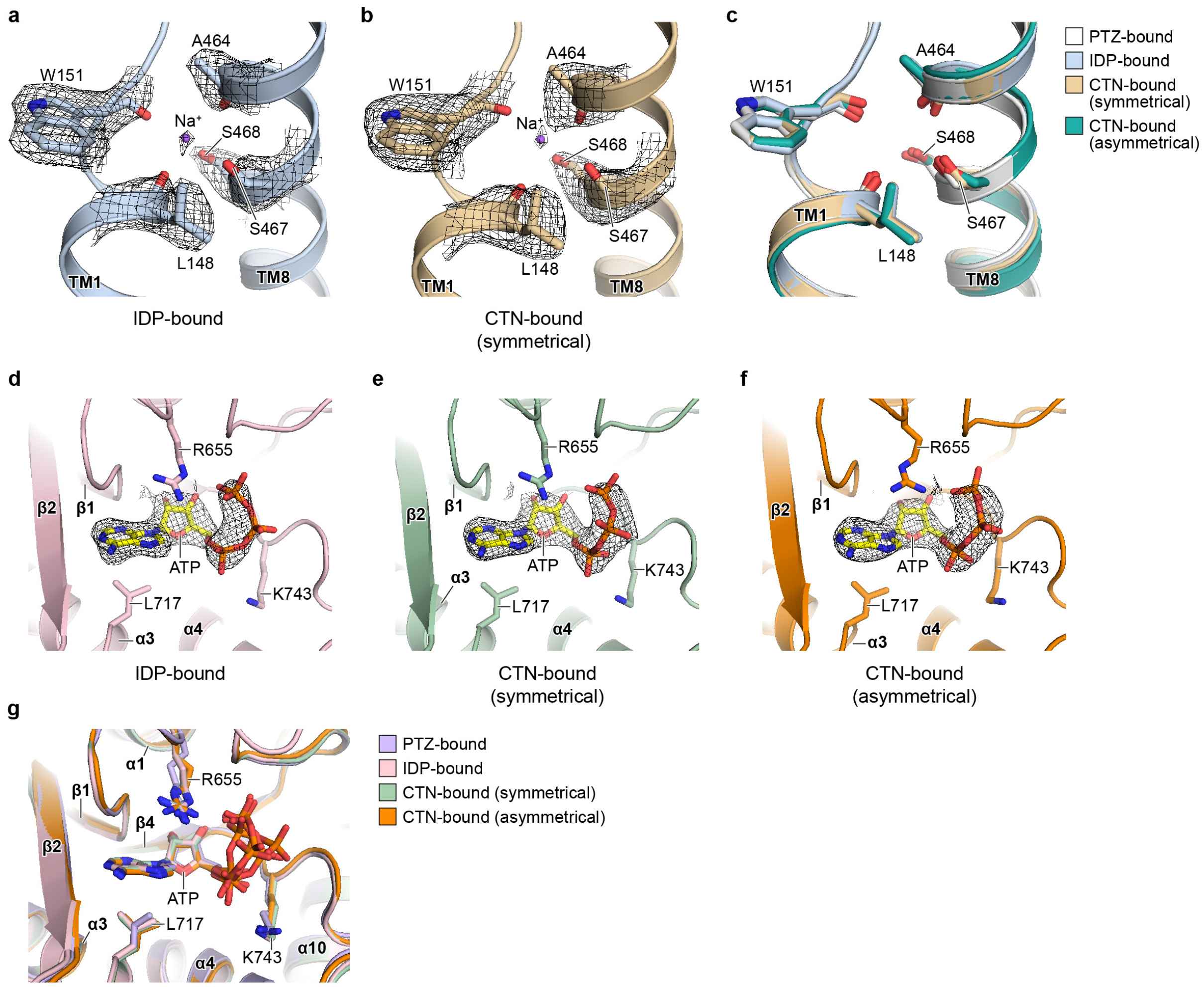

Supplementary Fig. 7 Comparisons of the Na$^+$-binding and nucleotide-binding sites. a. and **b.** Na$^+$-binding sites of the IDP-bound (light blue) and the symmetrical CTN-bound (tan) NCC, respectively. Na$^+$-coordinating residues are shown as sticks, and Na$^+$ ions are shown as purple spheres. Densities of Na$^+$ ions and coordinating residues are shown as meshes at contour levels of 9.5 σ and 7.5 σ in **a** and **b**, respectively. **c.** Comparisons of the Na$^+$-binding sites of NCC in complex with different thiazide diuretics. The PTZ-bound, IDP-bound, and symmetrical and asymmetrical (more CTD-interacting) CTN-bound structures are colored white, light blue, tan, and teal, respectively. **d.** to **f.** Nucleotide-binding sites of the IDP-bound (pink) and the symmetrical (green) and asymmetrical (orange) CTN-bound NCC, respectively. ATP (yellow) and key interacting residues are shown as sticks. Densities of ATP are shown as meshes at contour levels of 9 σ, 9 σ, and 10 σ in **d**, **e**, and **f**, respectively. **g.** Comparisons of the nucleotide-binding sites of NCC in complex with different thiazide diuretics. The PTZ-bound, IDP-bound, and symmetrical and asymmetrical CTN-bound structures are colored purple, pink, green, and orange, respectively. ATP molecules and key interacting residues are shown as color matched sticks. In **d-g**, the more TMD-interacting CTD subunits are used for analysis.

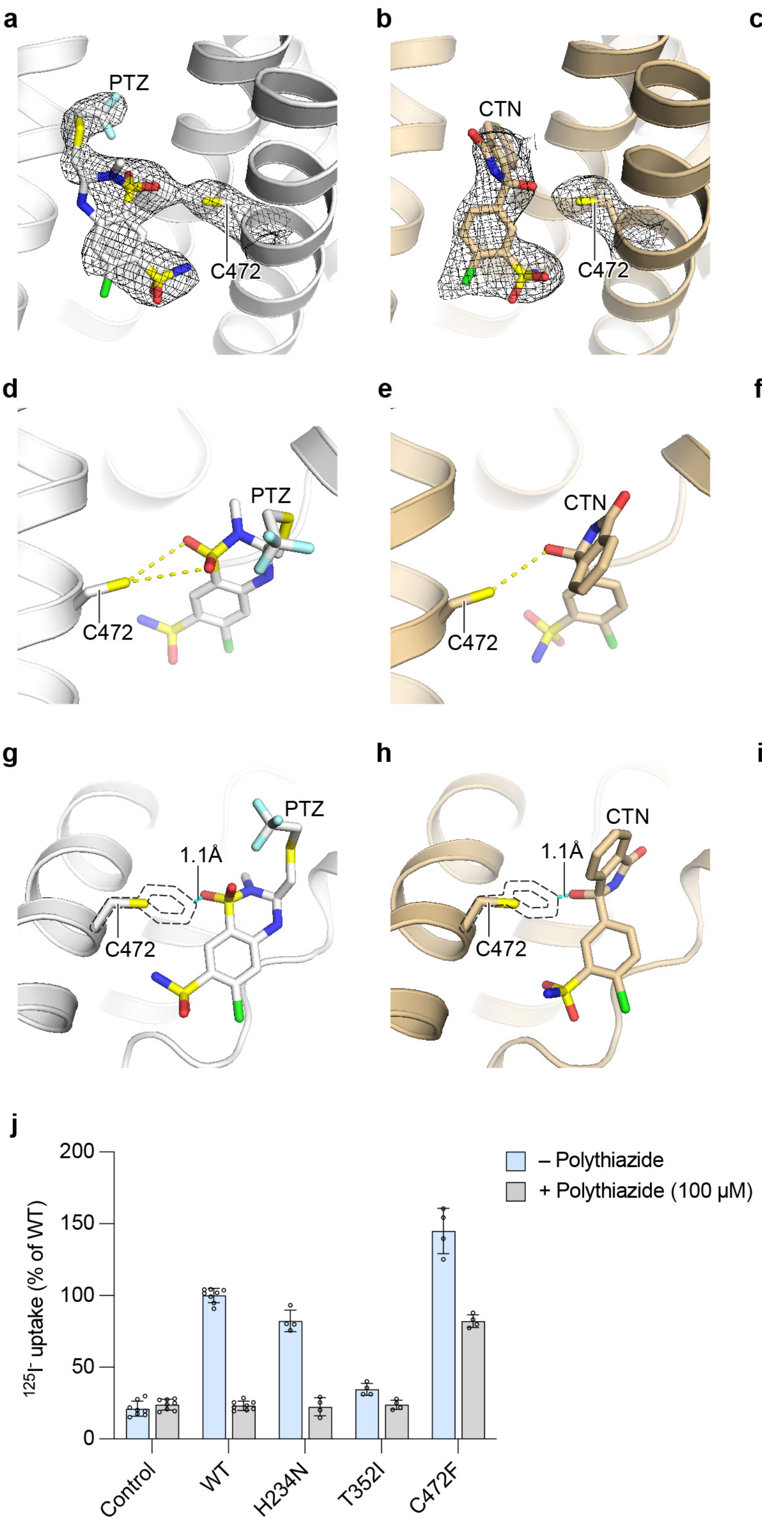

Supplementary Fig. 8 Ligand-specific interactions with C472. a. to **f.** Interactions between the side chain of C472 and different thiazide diuretics. The PTZ-, CTN-, and IDP-bound NCC structures are colored white, tan, and light blue, respectively. Bound thiazide diuretics and the side chain of C472 are shown as color matched sticks. In **a-c**, densities of bound thiazide diuretics and C472 are shown as meshes at a contour level of 8 σ. In **d-f**, polar interactions are shown as yellow dashed lines. **g.** to **i.** Steric

clashes with different thiazide diuretics introduced by the C472F substitution. The potential positions of the modeled side chains of the substituted phenylalanine residues are shown as dashed outlines. The potential distances between substituted phenylalanine residues and bound thiazide diuretics are labeled and highlighted with cyan dashed lines. **j.** Functional effects of substituting thiazide-interacting residues in hNCC with the corresponding eel βNCC-specific residues. The values are normalized to that of hNCC(WT) in the absence of polythiazide. Data are shown as mean ± s.d. (*n* = 8 independent experiments for control and hNCC(WT); *n* = 4 independent experiments for hNCC(H234N), hNCC(T352I), and hNCC(C472F)).

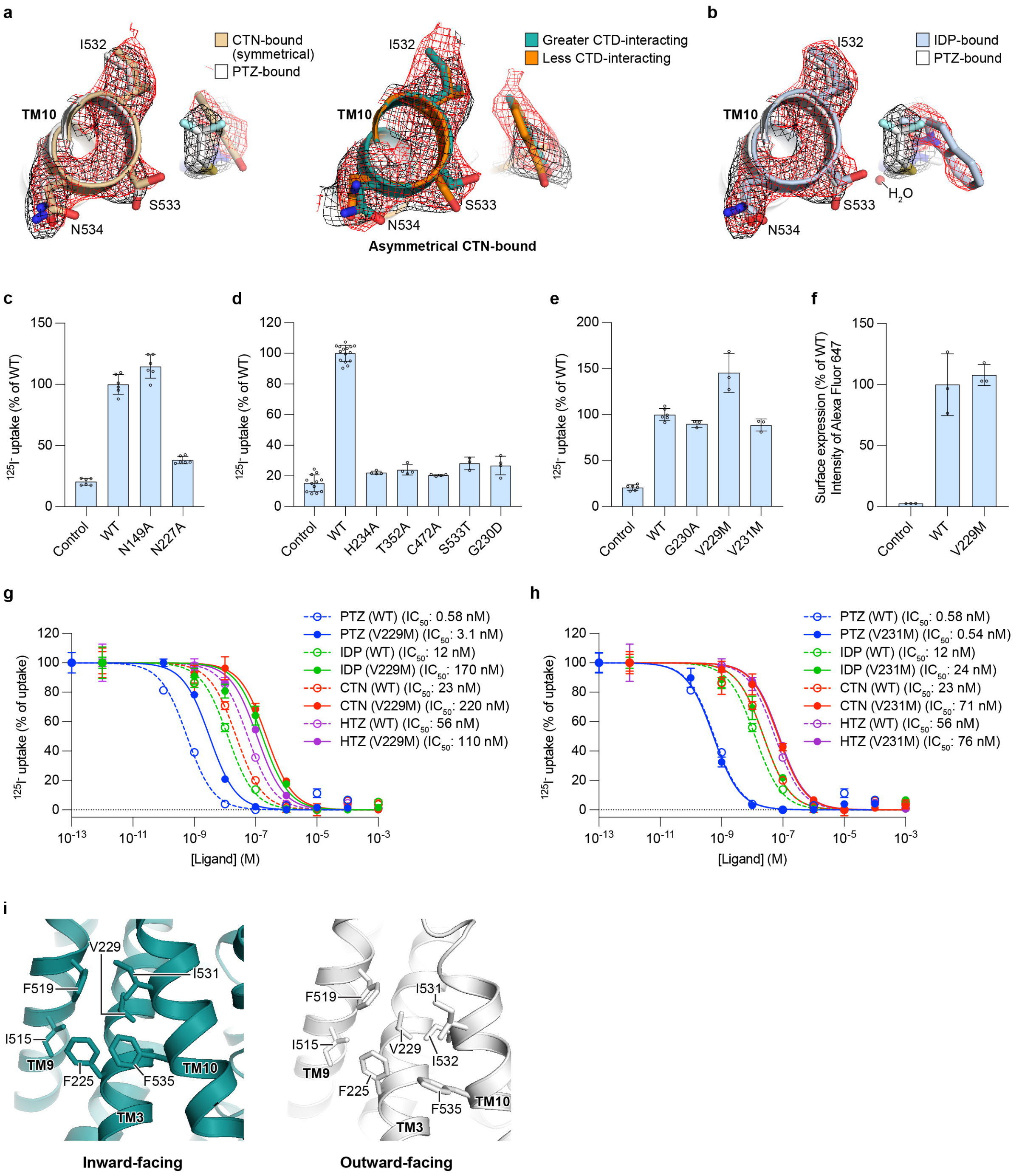

Supplementary Fig. 9 Ligand-specific orientations of S533 and differential effects of hNCC polymorphisms on sensitivities to different thiazide diuretics. a. and **b.** Comparisons of S533 side-chain orientations. The PTZ-bound, IDP-bound, CTN-bound (symmetrical), CTN-bound (asymmetrical, the TMD with greater CTD interaction), and CTN-bound (asymmetrical, the TMD with less CTD interaction) structures are colored white, light blue, tan, teal, and orange, respectively. Densities are shown as red (symmetrical CTN-bound (10 σ), asymmetrical CTN-bound with greater CTD-interaction (8 σ), and IDP-bound (9 σ)) or black (PTZ-bound (9 σ) and asymmetrical CTN-bound with less CTD-interaction (5 σ)) meshes. **c.** to **e.** Iodide-

uptake activities of several hNCC variants. The values are normalized to WT. Data are shown as mean ± s.d. In **c**, *n* = 6 independent experiments. In **d**, *n* = 12 and 15 independent experiments for control and WT, respectively. For H234A, T352A, C472A, and G230D, *n* = 4 independent experiments. For S533T, *n* = 3 independent experiments. In **e**, *n* = 6 independent experiments for control and WT. For G230A, V229M, and V231M, *n* = 3 independent experiments. One half of the control data are the same as in Supplementary Fig. 8j, and another half are the same as in Supplementary Fig. 9d. **f.** Surface expression of hNCC(V229M). The values are normalized to WT. Data are shown as mean ± s.d. (*n* = 3 independent experiments). **g.** Dose-response curves of different thiazide diuretics against hNCC(WT) or hNCC(V229M). Curves for hNCC(WT) and hNCC(V229M) are shown as dashed and solid lines, respectively. **h.** Dose-response curves of different thiazide diuretics against hNCC(WT) or hNCC(V231M). Curves for hNCC(WT) and hNCC(V231M) are shown as dashed and solid lines, respectively. In **g** and **h**, curves for chlorthalidone, indapamide, polythiazide, and hydrochlorothiazide are colored red, green, blue, and purple respectively, and data are shown as mean ± SEM (*n* = 3 independent experiments). WT data for PTZ, IDP, and CTN are the same as in Fig. 4b. **i.** Potential effects of the V229M polymorphism on hNCC conformational switches. Inward-facing (PDB: 8FHT) and outward-facing (PDB: 8FHO) conformations are colored teal and white, respectively. V229 and surrounding residues are shown as sticks.

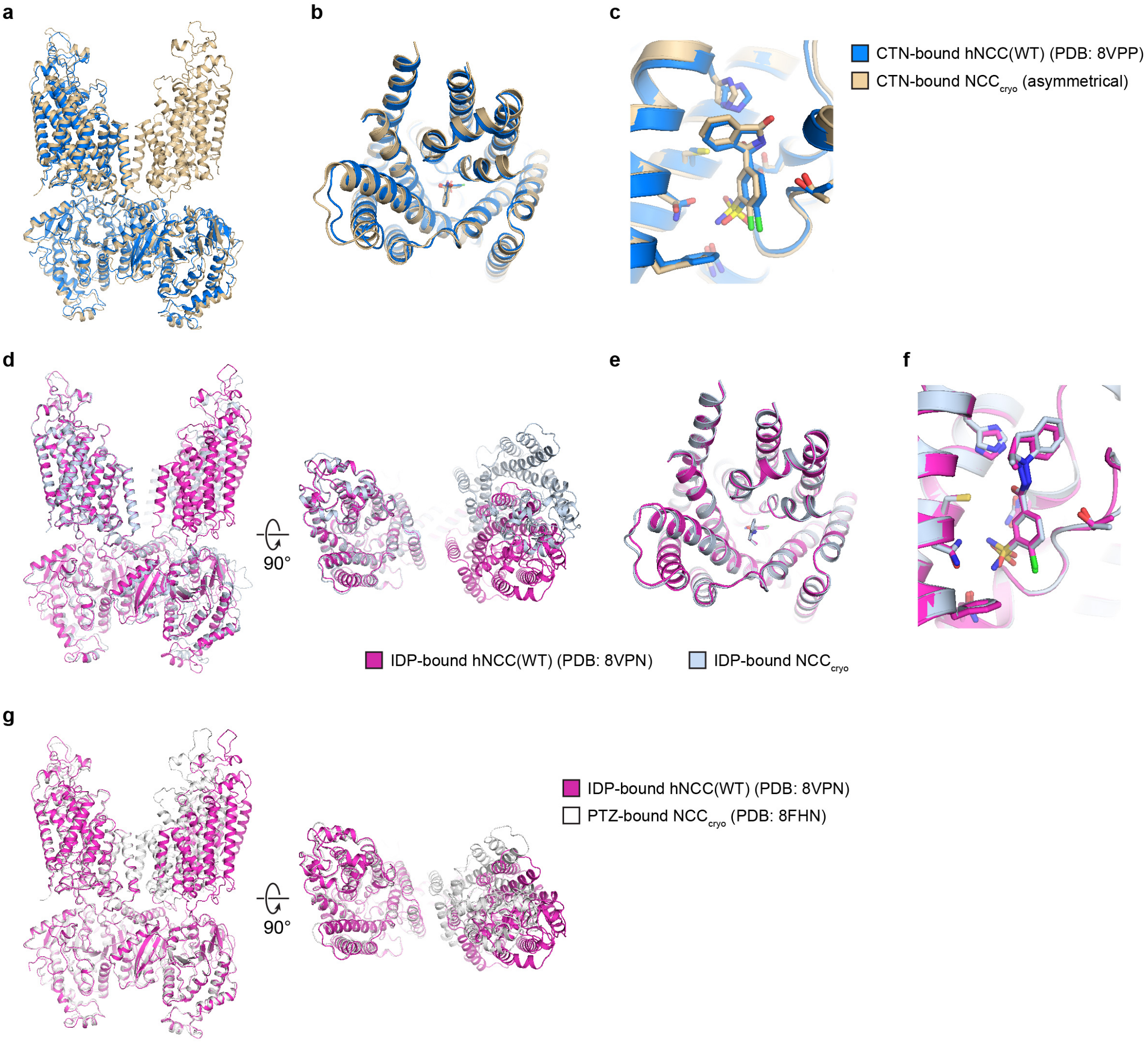

Supplementary Fig. 10 Comparisons of the thiazide diuretic-bound structures of NCC_{cryo} in nanodisc and hNCC in detergent micelle. a. to **c.** Comparisons of CTN-bound structures. CTN-bound hNCC (PDB: 8VPP) and NCC_{cryo} are colored blue and tan, respectively. The resolved TMD of 8VPP is aligned to the more CTD-interacting TMD subunit of NCC_{cryo}. **d.** to **f.** Comparisons of IDP-bound structures. IDP-bound hNCC (PDB: 8VPN) and NCC_{cryo} are colored magenta and light blue, respectively. The two structures are aligned based on the outward-facing (more CTD-interacting) TMD subunit of 8VPN. In **b** and **e**, ligands are shown as color matched sticks, and the cap domains are not shown for clarity. In **c** and **f**, ligands and key interacting residues are shown as sticks, and TM10-12 are not shown for clarity. **g.** Structural comparisons between IDP-bound hNCC (PDB: 8VPN) and PTZ-bound NCC_{cryo} (PDB: 8FHN). 8VPN and 8FHN are colored magenta and white, respectively. The two structures are aligned based on the more CTD-interacting TMD subunits.

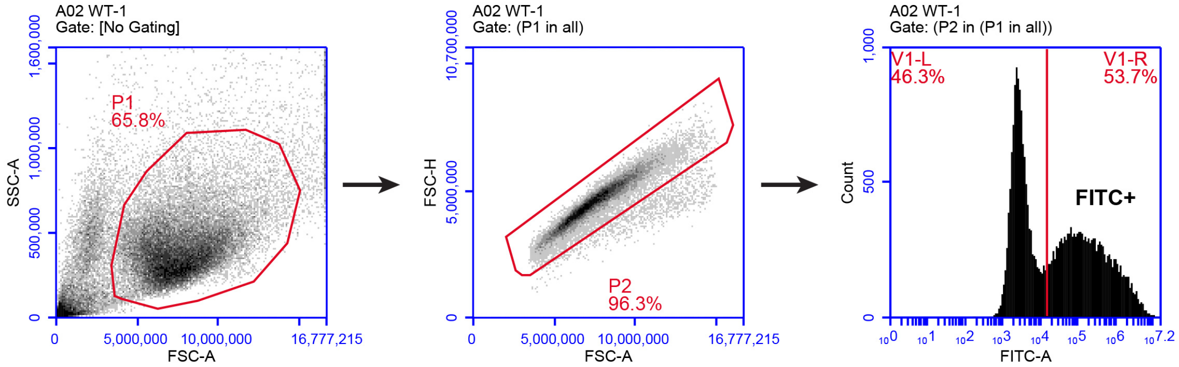

Supplementary Fig. 11 Flow cytometry gating strategy. Representative gating process used in measuring cell-surface expression. Intact HEK293S cells were first gated using the conventional FSC-A/SSC-A analyzing strategy. Single HEK293S cells were then gated using the standard FSC-A/FSC-H analyzing strategy. YFP-expressing single HEK293S cells, which were used for final Alexa 647 fluorescence intensity measurement, were then gated using the FITC channel as FITC+ cells.

Supplementary Table 1 Cryo-EM data collection, refinement and validation statistics

	Indapamide-bound NCC	Chlorthalidone-bound NCC	
**Data collection and processing**			
Magnification	105,000	105,000	
Voltage (kV)	300	300	
Electron exposure ($e^{-}/Å^2$)	50	50	
Defocus range (μm)	-1.2 to -2.0	-1.2 to -2.0	
Pixel size (Å)	0.86	0.86	
PDB code	9PIG	9PIF	9PIE
EMDB code	71666	71665	71664
Symmetry imposed	C2	C1	C2
Initial particle images (no.)	1,988,788	1,706,941	
Final particle images (no.)	446,219	96,877	103,392
Map resolution (Å)	2.79	3.24	3.01
FSC threshold	0.143	0.143	0.143
Map resolution range (Å)	2.47-12.31	2.80-46.10	2.63-35.19
**Refinement**			
Model resolution (Å)	2.9	3.4	3.2
FSC threshold	0.5	0.5	0.5
Model resolution range (Å)	275.2-2.79	275.2-3.24	275.2-3.01
Map sharpening *B* factor ($Å^2$)	-121.9	-103.8	-83.7
Model composition			
Non-hydrogen atoms	14038	13738	14004
Protein residues	1770	1736	1766
Ligands	8	5	8
B factors ($Å^2$)			
Protein	47.64	87.97	60.75
Ligand	55.68	79.31	69.58
R.m.s. deviations			
Bond lengths (Å)	0.004	0.003	0.004
Bond angles (°)	0.644	0.592	0.612
Validation			
MolProbity score	1.53	1.96	1.82
Clashscore	6.67	16.84	10.17
Poor rotamers (%)	0	0	0
Ramachandran plot			
Favored (%)	97.09	96.52	95.77
Allowed (%)	2.91	3.48	4.23
Disallowed (%)	0	0	0

Supplementary Table 2 hNCC polymorphisms affecting thiazide-interacting residues and their proposed potential effects on hNCC sensitivity to different thiazide diuretics.

Position	Variant (ID)	Proposed potential effects on NCC sensitivity to thiazide diuretics	Comment	Functional effects
N149	N149D (rs1207905694)	• Thiazide-type diuretics: decrease<br>• Indapamide: neutral<br>• Chlorthalidone: decrease	• N149 forms two hydrogen bonds with polythiazide and chlorthalidone. Therefore, N149D variant might be less sensitive to polythiazide and chlorthalidone because the side chain of aspartate may only be able to form one hydrogen bond.<br>• The pKa values of some thiazide-type diuretics are lower than 7.4, including chlorothiazide and trichlormethiazide, which corresponds to dissociation from the tertiary sulfamoyl group in the benzothiadiazine moiety[1]. For these acidic thiazide-type diuretics, the 2-position nitrogen may be negatively charged at pH 7.4. Therefore, the negative charge introduced by N149D, which is close to the 2-position nitrogen, might make NCC less sensitive to these acidic thiazide-type diuretics through electrostatic repulsion.<br>• N149 forms one hydrogen bond with indapamide. Therefore, N149D variation might have neutral effects on NCC sensitivity to indapamide because the side chain of aspartate may be able to form similar hydrogen-bonding interactions.	
N227	N227K (rs761240890)	Decrease	N227 is close to the shared free sulfamoyl groups of thiazide diuretics. Therefore, N227K variant might be less sensitive to all thiazide diuretics	Associated with Gitelman syndrome[2]

			because the longer side chain of lysine might lead to steric clashes with the shared free sulfamoyl groups.	
	N227S (rs773177438)	Decrease	N227 forms multiple hydrogen bonds with the shared free sulfamoyl groups of thiazide diuretics. Therefore, N227S variant might be less sensitive to thiazide diuretics because the side chain of serine may be too short to form hydrogen bonds with the shared free sulfamoyl group.	
	N227T (rs773177438)	Decrease	Similar to N227S, N227T variant might be less sensitive to thiazide diuretics because the side chain of threonine may be also too short to form hydrogen bonds with the shared free sulfamoyl group of thiazide diuretics.	
M233	M233V (rs387907469)	Decrease (May have neutral effects on hydrochlorothiazide)	M233 forms hydrophobic interactions with the bulky substituents of polythiazide, chlorthalidone, and indapamide. M233V variant might be less sensitive to thiazide diuretics with bulky substituents because the shorter side chain of valine cannot provide the same level of hydrophobic interaction as methionine. However, M233V might have a neutral effect on hydrochlorothiazide because it lacks a bulky substituent and is farther from M233.	Associated with Gitelman syndrome[3]
P349	P349A (rs1335601482)	Decrease (May have neutral effects on chlorthalidone and hydrochlorothiazide)	P349 forms hydrophobic interactions with longer thiazide diuretics, such as polythiazide and indapamide. P349A variant might be less sensitive to longer thiazide diuretics because the shorter side chain of alanine cannot provide the same level of hydrophobic interaction as proline. However, P349A variant might have neutral effects on shorter chlorthalidone and hydrochlorothiazide because they are farther from P349.	
	P349L (rs121909383)	Variable	P349L variant might be more sensitive to some thiazide diuretics because the longer side chain of leucine may provide more hydrophobic interaction than	Associated with Gitelman syndrome[4]

			proline. However, it also remains possible that P349L variation might generate steric clashes to some thiazide diuretics due to the longer side chain of leucine and make NCC less sensitive to those thiazide diuretics.	
T352	T352I (rs757876090)	• Thiazide-type diuretics: decrease<br>• Indapamide: neutral to decrease<br>• Chlorthalidone: neutral	• T352 forms a hydrogen bond with the 4-position nitrogen of the benzothiadiazine moiety of polythiazide. T352I variant might be less sensitive to thiazide-type diuretics because the longer and hydrophobic side chain of isoleucine might clash and cannot form hydrogen-bonding interactions with the 4-position nitrogen of the shared benzothiadiazine moiety.<br>• T352 is farther away from indapamide and chlorthalidone. Therefore, T352I variation might have neutral effects on NCC sensitivity to indapamide and chlorthalidone. However, T352 interacts indirectly with indapamide through a water molecule. Thus, T352I variation might slightly reduce NCC sensitivity to indapamide because the hydrophobic side chain of isoleucine cannot form similar indirect interactions with indapamide through a water molecule.	
N359	N359D (rs781030242)	Neutral	N359 forms polar interactions with the shared free sulfamoyl group of thiazide diuretics. N359D variation might have neutral effects on NCC sensitivity to thiazide diuretics because the side chain of aspartate is similar in size as asparagine and may also form similar polar interactions with the shared free sulfamoyl group.	Associated with Gitelman syndrome[5]
	N359K (rs181865675)	Decrease	N359 is close to the shared free sulfamoyl group of thiazide diuretics. Therefore, N359K variant might be less sensitive to thiazide diuretics	Associated with Gitelman syndrome[6]

			because the longer side chain of lysine may result in steric clashes with the shared free sulfamoyl group.	
	N359S (rs745709495)	Decrease	N359 forms polar interactions with the shared free sulfamoyl group of thiazide diuretics. Therefore, N359S variant might be less sensitive to thiazide diuretics because the shorter side chain of serine may not form similar polar interactions with the shared free sulfamoyl group.	
F536	F536L (rs748650798)	Decrease	F536 forms π-π stacking interactions with the benzene ring of the shared benzenesulfonamide moiety of thiazide diuretics. Therefore, F536L variant might be less sensitive to thiazide diuretics because the side chain of leucine cannot form similar π-π stacking interactions.	Loss-of-function mutation[7]
Y540	Y540C (rs2144719500)	Decrease	Y540 forms a halogen bond and/or Cl-π interactions with the 6-position chlorine group of polythiazide and the chlorine groups ortho to the free sulfamoyl groups of indapamide and chlorthalidone. Because a halogen or halogen-like group ortho to the shared free sulfamoyl group is common in thiazide diuretics, Y540C variant might be less sensitive to many thiazide diuretics because the shorter side chain of cysteine cannot form similar halogen-bonding or Cl-π interactions.	Associated with Gitelman syndrome[8]

Supplementary References:

1 Goto, S., Odawara, Y., Nakano, M. & Araki, Y. [Stability and serum albumin binding of diuretics in aqueous solution (author's transl)]. *Yakugaku Zasshi* **98**, 236-241 (1978).

2 Tavira, B. *et al.* A labor- and cost-effective non-optical semiconductor (Ion Torrent) next-generation sequencing of the SLC12A3 and CLCNKA/B genes in Gitelman's syndrome patients. *J Hum Genet* **59**, 376-380 (2014).

3 Wang, F., Shi, C., Cui, Y., Li, C. & Tong, A. Mutation profile and treatment of Gitelman syndrome in Chinese patients. *Clin Exp*

Nephrol **21**, 293-299 (2017).
4 Simon, D. B. *et al.* Gitelman's variant of Bartter's syndrome, inherited hypokalaemic alkalosis, is caused by mutations in the thiazide-sensitive Na-Cl cotransporter. *Nat Genet* **12**, 24-30 (1996).
5 Miao, Z. *et al.* Coexistence of normotensive primary aldosteronism in two patients with Gitelman's syndrome and novel thiazide-sensitive Na-Cl cotransporter mutations. *Eur J Endocrinol* **161**, 275-283 (2009).
6 Qin, L. *et al.* Identification of five novel variants in the thiazide-sensitive NaCl co-transporter gene in Chinese patients with Gitelman syndrome. *Nephrology (Carlton)* **14**, 52-58 (2009).
7 De Jong, J. C. *et al.* Functional expression of mutations in the human NaCl cotransporter: evidence for impaired routing mechanisms in Gitelman's syndrome. *J Am Soc Nephrol* **13**, 1442-1448 (2002).
8 Vargas-Poussou, R. *et al.* Spectrum of mutations in Gitelman syndrome. *J Am Soc Nephrol* **22**, 693-703 (2011).

Source images of the SDS-PAGE gels

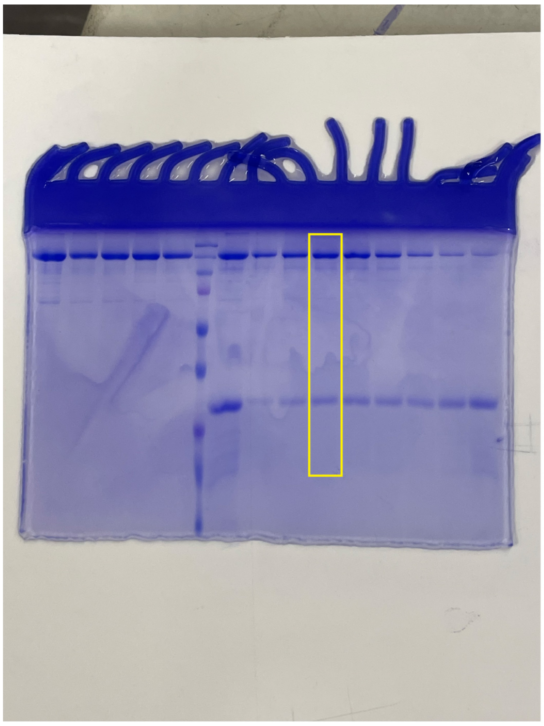

Raw image of Supplementary Fig. 2a

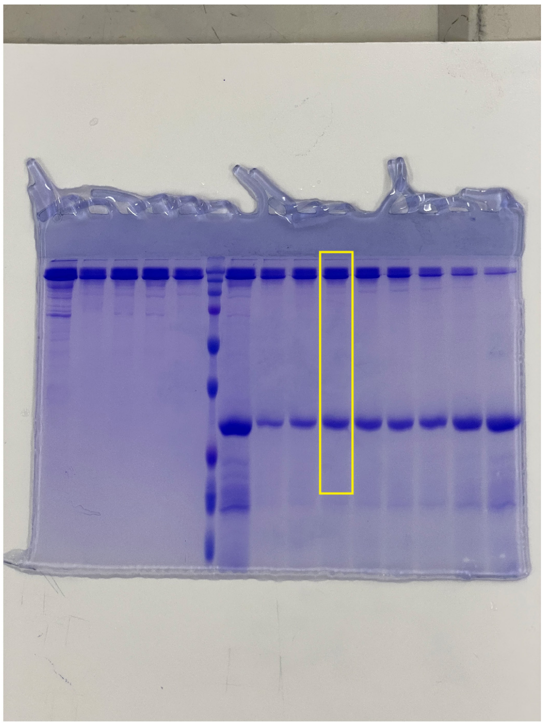

Raw image of Supplementary Fig. 2b
